# CHK1 expression in Gastric Cancer is modulated by p53 and RB1/E2F1: implications in chemo/radiotherapy response

**DOI:** 10.1038/srep21519

**Published:** 2016-02-12

**Authors:** J. Bargiela-Iparraguirre, L. Prado-Marchal, M. Fernandez-Fuente, A. Gutierrez-González, J. Moreno-Rubio, M. Muñoz-Fernandez, M. Sereno, R. Sanchez-Prieto, R. Perona, I. Sanchez-Perez

**Affiliations:** 1 Dpto.Bioquímica. Fac. Medicina. Instituto de Investigaciones Biomédicas Madrid CSIC-UAM; Madrid, Spain; 2 The Royal Veterinary College. University of London; London, UK; 3 Medical Oncology Department, Infanta Sofía University Hospital, San Sebastian de los Reyes, Madrid, 28702; Spain; 4 IMDEA-Food Institute, CEI UAM+CSIC, Madrid, Spain; 5 Pathology Department, Infanta Sofía University Hospital, San Sebastián de los Reyes, 28702, Madrid; 6 Unidad de Medicina Molecular, laboratorio de Oncología, CRIB/FPCYT C-LM. Universidad de Castilla-La Mancha, Av. Almansa 14, 02006, Albacete, Spain; 7 Unidad asociada de Biomedicina UCLM-CSIC, Arturo Duperier 4, Madrid Spain; 8 CIBER for Rare Diseases (CIBERER); Valencia, Spain; 9 Biomarkers and Experimental Therapeutics Group; IdiPAZ; University Hospital La Paz; Madrid, Spain

## Abstract

Radiation has a limited but relevant role in the adjuvant therapy of gastric cancer (GC) patients. Since Chk1 plays a critical function in cellular response to genotoxic agents, we aimed to analyze the role of Chk1 in GC as a biomarker for radiotherapy resistance. We analyzed Chk1 expression in AGS and MKN45 human GC cell lines by RT-QPCR and WB and in a small cohort of human patient’s samples. We demonstrated that Chk1 overexpression specifically increases resistance to radiation in GC cells. Accordingly, abrogation of Chk1 activity with UCN-01 and its expression with shChk1 increased sensitivity to bleomycin and radiation. Furthermore, when we assessed Chk1 expression in human samples, we found a correlation between nuclear Chk1 accumulation and a decrease in progression free survival. Moreover, using a luciferase assay we found that Chk1’s expression is controlled by p53 and RB/E2F1 at the transcriptional level. Additionally, we present preliminary data suggesting a posttranscriptional regulation mechanism, involving miR-195 and miR-503, which are inversely correlated with expression of Chk1 in radioresistant cells. In conclusion, Chk1/microRNA axis is involved in resistance to radiation in GC, and suggests Chk1 as a potential tool for optimal stratification of patients susceptible to receive adjuvant radiotherapy after surgery.

Gastric cancer (GC) is the fourth most common human malignant disease worldwide due to its frequency and high rate of mortality^1^,^2^. Classical adjuvant treatment in GC is based on MacDonald´s protocol including a combination of 5-Fluoruracil (5-FU) and radiation (IR) in stage IB-IVA patients. This regimen demonstrated an increase of progression free survival (PFS) and overall survival (OS)^3^,^4^. However, this combination is usually associated with increased, severe toxicity^3^. Thus, identification of those patients more prone to benefit from adjuvant radiation and 5-FU after curative surgery for GC to counteract such toxicity is an unmet need^5^,^6^.

Radiation and chemotherapy routinely used to treat cancer do cause a variety of DNA lesions, which in turn activate DNA damage response (DDR)^7^. Checkpoint Kinase 1 (Chk1), a key effector in DDR, is a multifunctional Ser/Thr kinase protein highly conserved through evolution^8^ and represents a crucial component in all cell cycle checkpoints. Chk1 activation must be finely regulated to ensure its adequate activity. The major known to date mechanism controlling Chk1 regulation is the phosphorylation of specific residues Ser^317^ and Ser^345^ (both in the C-terminal domain), which leads to catalytic activation. These reactions are catalyzed by ATR and ATM kinases^7^,^8^,^9^. Deregulation of Chk1 expression has been previously described in cancer, i.e. Chk1 is overexpressed^7^,^10^,^11^,^12^ and has been correlated with radiotherapy resistance in some cancer types such as ovarian cancer^13^, nasopharyngeal carcinoma^14^ and lung cancer^15^. Accordingly, inhibition of Chk1 increases sensitivity to chemo-radiotherapy in multiple tumor models^16^,^17^. Given this apparent relationship between Chk1 expression and resistance to certain therapies, some groups have made an effort to evaluate Chk1 as a novel target to improve cancer therapy^18^ in patients that have been previously exposed to ionizing agents. Besides the well known mechanisms regulating both activation and expression of Chk1 that have already been described^10^,^19^,^20^,^21^,^22^,^23^, Chk1 is also regulated at the post-transcriptional level by microRNAs (miRNAs)^24^,^25^ which are key regulators of tumor growth and response to chemotherapy^26^,^27^,^28^. In fact, some studies have already begun to identify miRNAs involved in sensitizing or causing resistance to chemotherapy, thus providing potential new targets and mechanisms as optimized treatment options^29^,^30^,^31^.

Previous studies from our laboratory suggested that Chk1 levels could be used as a predictive biomarker of therapeutic response in colon cancer^32^. Furthermore, we demostratred that E1A upregulates Chk1 and this correlates with glioblastoma radiosensitivity^33^. Here, we prove that Chk1 is overexpressed in a disseminated GC cell line, MKN45. We have shown that the inhibition of Chk1 results in increased IR sensitivity. Accordingly, we observed nuclear accumulation of Chk1, which correlates with a lower PFS period, in a cohort of patients treated with IR. The results we present here, suggest that Chk1 protein levels are controlled at the transcriptional level mainly through RB1/E2F1 and our preliminary data suggest that at the posttranscriptional level they are likely regulated by miRNA-195 and -503. We conclude that Chk1 is responsible for radiation resistance in GC, and suggest Chk1 as a potential biomarker for the optimal stratification of patients susceptible to receive adjuvant radiotherapy after surgery.

## Results

### Increased Chk1 expression correlates with resistance to genotoxic agents in GC cells

**Figure 1:**
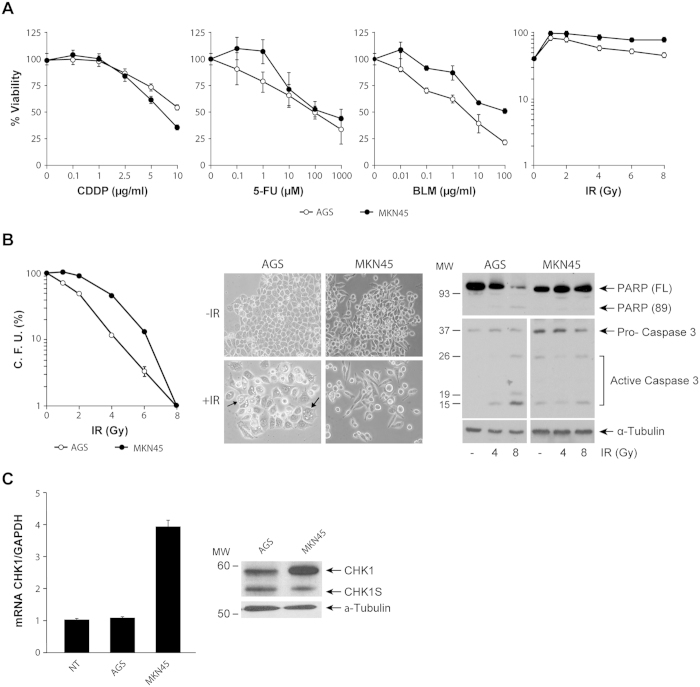
CHK1 mRNA levels are high in GC cells resistant to radiation (IR) and bleomycin (BLM). (**A**) Survival of AGS and MKN45 cells after CDDP, 5-FU, BLM or IR treatment. AGS (○) and MKN45 (●) cells were treated with increasing amounts of CDDP (0–10 μg/ml), 5-FU (0–1000 μM), BLM (0–100 μg/ml) or IR (0–8 Gy). 48 h after treatment, the percentage of viable cells was quantified by the MTS method. Data represent the mean values obtained in two experiments performed in quadruplicate. (**B**) Clonogenic assay in AGS and MKN45 cells 13 days after irradiation with different doses of Gy (0–8); the graph shows the percentage of CFU (colony forming units). Representative images of AGS and MKN45 cells, 13 days after irradiation (4 Gy) are shown. The arrows point to abnormal morphology in AGS cells. Cleavage of PARP-1 was detected by western blot (WB) in cells harvested 24 h after 4 and 8 Gy IR. Activation of Caspase 3 was detected in the same extracts as above, running under the same experimental conditions. (Full length blot is included in supplementary information). α-Tubulin was used as a loading control. (**C**) RT-QPCR analysis of *CHK1* expression in asynchronous cultures of AGS and MKN45 cells. The graph shows the relative levels of CHK1’s mRNA compared to normal tissue (NT), and using GAPDH as endogenous control. WB for both Chk1 isoforms (Chk1 and Chk1-S) in whole cell extracts from asynchronous cultures of AGS and MKN cells. α-Tubulin was used as a loading control.

We studied the sensitivity of the two most common human gastric adenocarcinoma cell lines (AGS and MKN45) to antitumoral agents: cisplatin (CDDP), 5-FU, the radiomimetic agent Bleomycin (BLM) and IR. Dose response curves using this set of drugs showed that after 48 hours of treatment, MKN45 cells are more resistant than AGS cells to both BLM or IR treatment (Fig. 1A), with this increased resistance more evident after BLM treatment. To corroborate this result we conducted a clonogenic assay, and observed that the percentage of colony formation was higher on MKN45 cells than on AGS after 4 Gy irradiation (i.e: 89,6% vs 48.7% respectively) (Fig. 1B). Similarly, when treated with BLM, AGS cells were unable to form colonies at all (Supplementary Fig. 1A); Furthermore, we also observed morphological changes characteristic of cell death in irradiated AGS cells, which were completely absent in MKN45 cells (Fig. 1B). To support this finding we irradiated cells with 4 and 8 Gy for 24 hours. We observed cleavage of PARP, and also a decrease in the full-length form of this protein in AGS cells in a dose dependent manner. However, in MKN45 we only observed basal levels of PARP with no change in proteolysis. Furthermore, the universal marker of apoptosis, activation of Caspase 3, is also observed in the AGS cell line, with particularly strong activity after 8Gy treatment (Fig. 1B).

Given that Chk1 is one of the main effector proteins on the response to IR exposure, we examined the expression levels of Chk1 in MKN45 and AGS cells by RT-QPCR and western blot (WB). Our results showed an increase on Chk1 mRNA and protein levels in both MKN45 and AGS cells when compared to normal tissue. Two different isoforms of Chk1 have been described^34^ and our WB analysis indicated that the more abundantly expressed isoform in MKN45 is the classic Chk1 with higher molecular weight (Fig. 1C). We confirmed this result by analyzing the expression of this gene, using public data available on Oncomine database (http//:oncomine.org) (Supplementary Fig. 1B). Our semi-quantitative PCR analysis confirmed the expression of both full-length and short forms of Chk1 in these two cell lines (Supplementary Fig. 1C). Altogether, these results suggest that elevated levels of Chk1 in GC cells correlate with a lack of apoptotic response to IR or BLM treatment.

### Chk1 inhibition reduces radioresistance in GC cell lines

**Figure 2:**
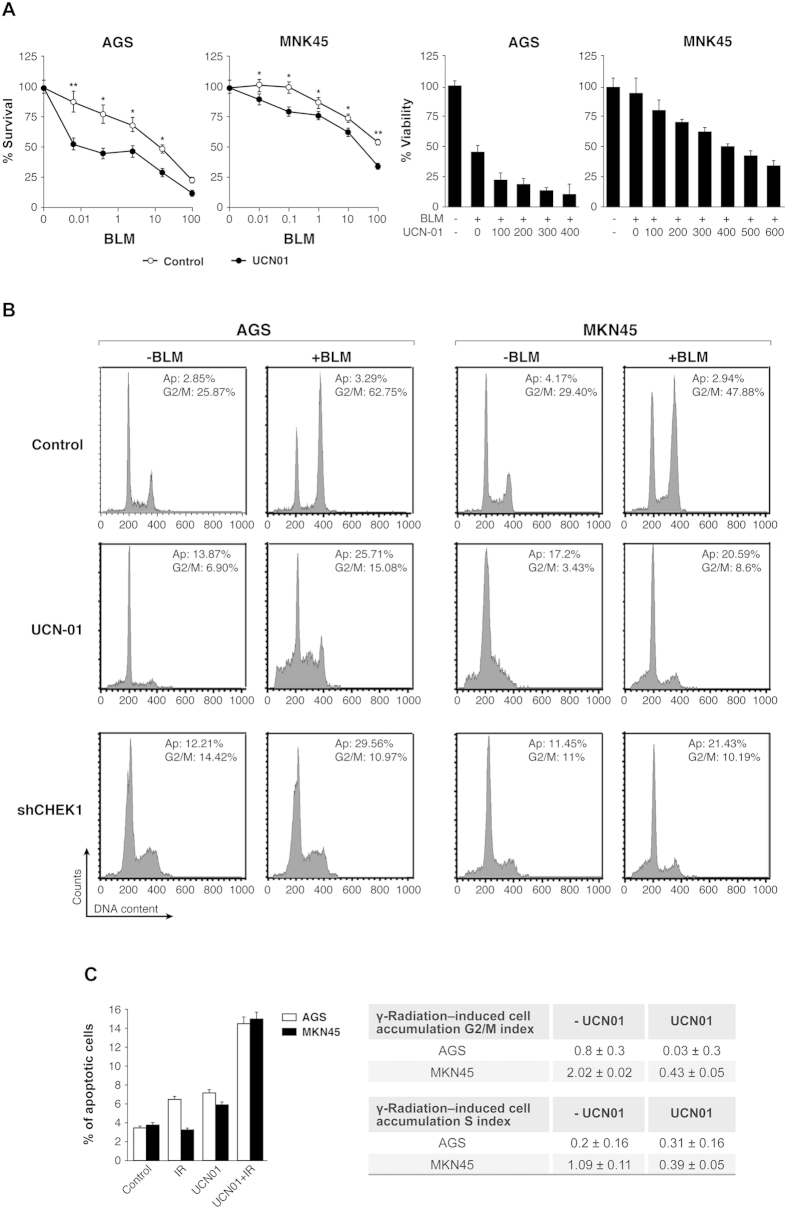
Chk1 inhibition sensitizes GC cells to BLM or IR. (**A**) Survival rates in AGS and MKN45 cell lines, after treatment with BLM (0–100 μg/ml) and in the presence (●) or absence (○) of 100 nM UCN-01. Histogram: Viability percentage for AGS and MKN45 cells treated with increasing doses of UCN-01 (0–600 nM) in the presence or absence of BLM (3 μg/ml and 10 μg/ml for AGS and MKN45 cells respectively). (**B**) Cell cycle profile after inhibition of Chk1 by treatment with the inhibitor UCN-01 (100 nM for AGS or 300 nM for MKN45 cells) or after silencing Chk1’s expression by transient transduction with a lentivirus carrying shRNA- Chk1 for 72 hours. One hour after, cells were treated with vehicle or with BLM (3 ug/ml) for 24 hours. Plots are representative of an experiment performed twice in duplicate. AP: Apoptotic Cells, G2/M: cells in G2 or Mitosis. (**C**) Percentage of apoptotic cells in both cell lines after irradiation, UCN-01 treatment and UCN-01 plus IR. Table containing G2/M and S accumulation index in both cell lines after IR and with or without UCN-01 treatment.

To confirm the effect of Chk1 on survival to double strand breaks (DBS) induced by BLM or IR treatment in GC cells, we inhibited Chk1 activity by using the chemical inhibitor UCN-01 prior to treatment. Inhibition of Chk1 with 100 nM UCN-01 (no toxicity observed) increased mortality rate in combination with BLM treatment (0–100 μg/ml). Addition of UNC-01, significantly decreased IC_50_ in both cell lines: from 2.45 to less than 1 μg/ml in AGS and from over 100 to 11 μg/ml in MKN45 cells (Fig. 2A-Left panel). On the other hand, treatment with BLM using 3 μg/ml for AGS cells and 10 μg/ml for MKN45 followed by UCN-01 (0–600 nM) also sensitized both cell lines (Fig. 2A- Right panels); however the dose of UCN-01 required to increase sensitivity to BLM in MKN45 cells was highly toxic (>400 nM). These results suggest a potential synergistic or additive effect upon the combination treatment of BLM with UCN-01. We therefore used the Combination Index (CI) equation method developed by Chou-Talalay^35^ using the CalcuSyn program to study combinational synergistic effects. Our studies revealed that in AGS cells both drugs exhibit a synergistic effect (CI < 1) at all the UCN-01 doses tested in combination with BLM. By contrast, when using MKN45 cells we only observed that synergistic effect (CI < 1) at very high toxic doses of UCN-01. Furthermore, in this cell line our drugs exerted an antagonistic effect (CI > 1) at lower doses. To further assess the contribution of Chk1 in the response to therapy, we downregulated Chk1 by pharmacological agents (UCN-01) or silenced it using shRNA lentiviral particles. Next, we analyzed the cell cycle profile after challenge with BLM in cells that had been pre-treated with UCN-01 or after interference on Chk1’s expression. We observed a drastic abrogation of the G2/M checkpoint after both treatments, which was even more dramatic when Chk1 is interfered with by our lentivirus (Fig. 2B). Furthermore, we found that the percentage of apoptotic cells was increased in both cell lines after the combination treatment (UCN-01+ IR) (Fig. 2C- Graph). The analysis of the G2/M and S indexes indicated that UCN-01 abolishes the G2/M and Intraphase S checkpoint in GC cells further supporting the contribution of Chk1 to radiation resistance (Fig. 2C- Table). To confirm that UCN-01 inhibits Chk1, we monitored Chk1 autophosphorylation on Ser296 as previously described^36^ (Supplementary Fig. 2A). As a control confirming Chk1’s silencing we observed that after 72 hours, AGS cells showed almost complete abolishment of Chk1 mRNA expression and around a 60% reduction for MKN45 cells (Supplementary Fig. 2B). Taken together, the data presented here suggest that Chk1 depletion results in enhanced sensitivity, lower survival rates following exposure to radiation, and altered G2/and intra-S checkpoint responses to DSBs induced damage.

### Transcriptional regulation of Chk1 in GC cells

**Figure 3:**
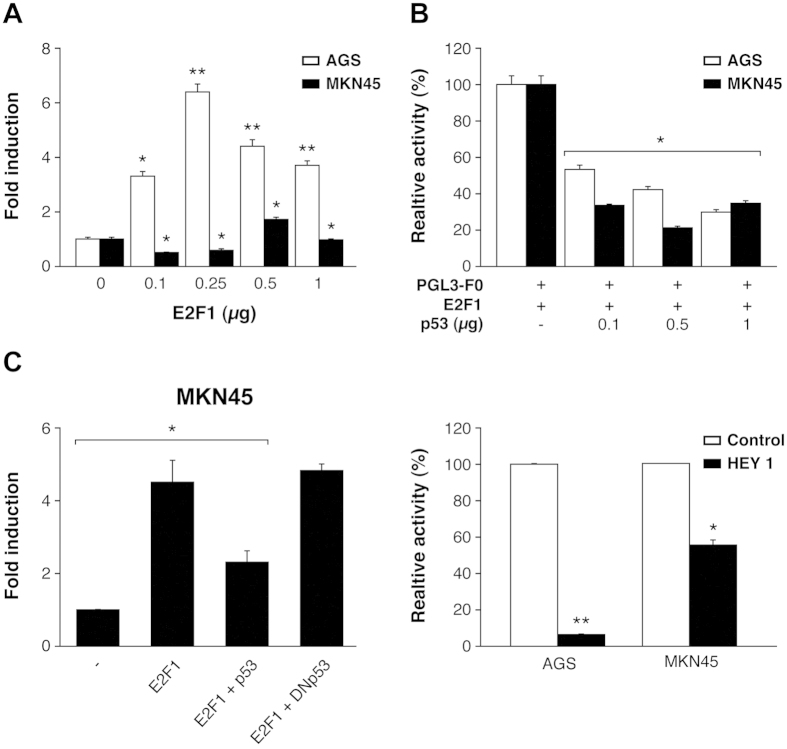
Chk1’s promoter activity is regulated by E2F1 and p53 in GC cell lines. (**A**) Cells were transfected with increasing doses (0–1 μg) of an E2F1 expression vector (pCMV-E2F1), the corresponding empty control expression vector, and 200 ng of pGL3-F0. The graph shows activity levels, relative to that of PGL3-F0. (**B**) AGS and MKN45 cells were transfected with 200 ng of PGL3-F0, 500ng pCMV-E2F1 and increasing doses of p53’s expression vector (0–1 μg). Results are presented as activity level, relative to that of the empty pGL3-Luc reporter in the presence of E2F1. (**C**) Left graph: MKN45 cells were transfected with 200 ng pGL3-F0 and expression vectors for E2F1 (250 ng), E2F1 plus p53 or E2F1 plus DNp53. Right graph: Both MKN45 and AGS cells were transfected with 200 ng pGL3-F0 and expression vectors for E2F1 (250 ng) or HEY1 (200 ng). All data are presented as the average of at least three independent experiments assayed in triplicate ± SEM. p < 0,05 versus empty vector control (Student’s *t* test).

**Figure 4:**
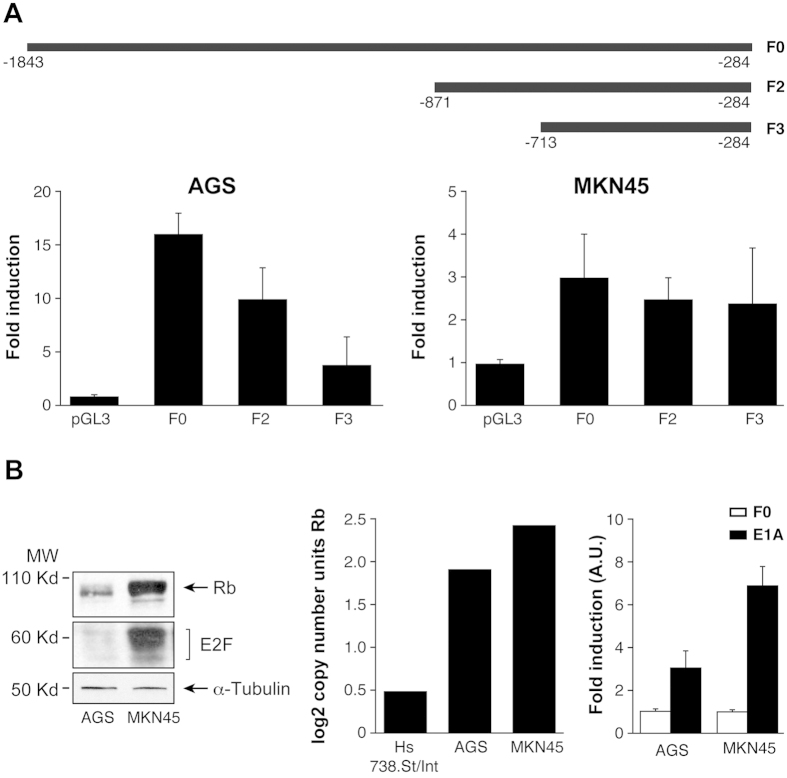
The RB1/E2F1 axis controls activation of Chk1 promoter. (**A**) Schematic representation of the constructs used in the transfection experiments. AGS and MKN45 were transfected with 250 ng of the indicated construction, and luciferase activity was measured 24 h later. The histograms show relative activity normalized to the empty vector in both AGS and MNK45 cells. (**B**) Expression of both RB1 and E2F1 was detected by WB using specific antibodies in AGS and MKN45 cells. α-Tubulin was used as loading control. The experiments were repeated three times with similar results. The graph on the center represents relative expression level (log 2-copy number) of RB1 in control (Hs 738St/Int) and tumoral AGS and MKN45 cells obtained from the Oncomine database. The graph on the right shows luciferase activity measurements in AGS and MKN45 cells after transfection with 250 ng of F0 and 100 ng of PCEFL-E1A. The data are presented as activity relative to the values found for the empty vector in transfected cells. The experiment was performed twice in triplicate.

To study whether high levels of Chk1 occur due to alterations at the transcriptional level, we cloned a fragment of Chk1 corresponding to the 5′ Flanking region of the gene, (−1823 −284, as predicted by the Transfac tool) which contains the transcription factor (TF) and binds to CHK1’s promoter (Supplementary Fig. 3A), in the pGL3-Basic enhanced luciferase plasmid. We detected luciferase activity after transient transfection in AGS and MKN45 cells; this occurred in both cell lines in a DNA- dose dependent manner, confirming that the generated construct is functional (Supplementary Fig. 3B). First, we focused on p53 and E2F1. E2F1 expression in AGS cells reached a 6-fold increase over pGL3-F0; however, only a 2-fold increase was observed in MKN45 cells (Fig. 3A). Next, we analyzed if both E2F1 and p53 cooperate in modulating Chk1 expression in GC cells. We verified that both cells lines are p53 wild type^37^,^38^ and also performed a WB in order to evaluate the status of p53 in our specific experimental conditions, and our results indicated that p53 activation is equivalent in both cell lines. We found a transient activation of p53 4 h after IR (8 Gy) which returns to basal levels 24 hours after. We did not observe differences in p53 basal levels either (Supplementary Fig. 3C). To analyze the contribution of E2F1 and p53 in Chk1 expression, we performed an experiment in which cells were co-transfected with E2F1 (250 ng) and pGL3-F0 (200 ng) expression plasmids and increasing doses of the p53 (0–1 μg) expression vector. Our results showed that p53 was able to inhibit the transcriptional activity induced by E2F1 expression in GC cells, in a dose-dependent manner (Fig. 3C). We also tested the Dominant Negative (DN) form of p53 that was indeed able to revert the p53-dependent down-regulation of Chk1 when co-transfected with E2F1 and pGL3-F0, which further supported the involvement of p53 in Chk1 promoter regulation (Fig. 3D). To confirm that p53 regulates Chk1 promoter *in vivo*, cells were co-transfected with a plasmid encoding the Hey1 protein, which is an activator of p53^39^ and pGL3-F0. Hey1 completely repressed the activity of Chk1 promoter after E2F1 expression in AGS cells and strongly inhibited it (50%) in MKN45 cells (Fig. 3D). To verify that these TFs control Chk1’s expression in AGS cells, we quantified Chk1’s mRNA levels under the above conditions. Our results suggest that E2F1 increases Chk1 expression and that activation of p53 reduces it (Supplementary Figure 3D). These results suggest that transcriptional regulation in these cells could be differentially modulated in GC adenocarcinoma through changes on E2F1 protein levels. The “*in silico*” analysis of the promoter sequence, revealed the presence of different E2F1 binding sites; therefore, we performed different deletions of the 5′ UTR (F2) and (F3), taking into account the putative TF binding sites for E2F. F2 and F3 are fragments that contain two and one E2F binding sites respectively (Fig. 4A). We found a drastic decrease in the transcriptional activation in AGS cells dependent on the number of E2F1 binding sites; however, no significant differences were observed in MKN45 cells. This suggests that the two E2F1 binding sites located in the area between −1843 −1201 pb (lost in F2 and F3) play a pivotal role in the induction of the promoter activity, especially in the AGS cell line, which suggests that other mechanisms are involved in the regulation of *CHK1* mRNA levels in MNK45 cells (Fig. 4A).

To further explore the mechanism involved in Chk1 expression by E2F1 and to gain insight into the differences found in E2F1 regulation between these cell lines, we studied the basal expression of RB1 and E2F1 proteins in each cell line. Our results indicate that levels of both RB1 and E2F1 are higher in MKN45 than in AGS cells (Fig. 4B). The expression of the RB gene through the Oncomine database (http//:oncomine.org), confirmed our finding of high levels of RB1 mRNA in MKN45 cells (Fig. 4B middle graph). We took advantage of the ability of adenovirus E1a protein to bind and block this tumor suppressor gene *RB*^40^,^41^. Transfection of a plasmid encoding the E1a protein in both cell lines, resulted in a clear increase on the activity of Chk1 promoter, which was more dramatic in MKN45 cells (Fig. 4B- right graph). These results indicate that the lack of transcriptional activation of Chk1 promoter in MKN45 cells and to a lower extent in AGS cells, could be due to effects exerted by the RB1 protein through binding to the TF E2F1.

The above results do not completely justify the high level of *CHK1* messenger in MKN45 cells. Therefore, we investigated if the increase on *CHK1* mRNA level in MKN45 cells is a result of post-transcriptional regulation. To investigate this possibility, we treated AGS and MKN45 cells with the transcriptional inhibitor Actinomycin D (Act D). CHK1 mRNA level was reduced 9 hours after Act D treatment in AGS cells (0,75-fold). However, in the MNK45 cell line, mRNA was accumulated at the same time (2,66-fold) (Supplementary Fig. 4A). These results suggest that CHK1 mRNA stability is regulated at post-transcriptional level in MKN45 cells.

Therefore, we decided to investigate the expression of microRNAs in samples from human GC cells in order to find miRNA candidates to regulate Chk1 expression. To this end, we analyzed the gene expression dataset GSE30070^42^, and found that the levels of microRNA predicted by the online software TARGETSCAN for *CHK1* were significantly different between cancer and control samples (Supplementary Fig. 4B). From the predicted miRNAs that target *CHK1*, we investigated the potential role of miR-195 and miR-503 in the regulation of the stability of *CHK1* mRNA. miR-195 belongs to the big miR-15 microRNA family. This family has been recently described as radiosensitivity enhancer on breast cancer by targeting *CHK1*^43^ and miR-503 has been previously shown to target *CHK1*^44^,^45^. We then used real-time PCR to detect the expression of miR-195/503 in AGS and MKN45 cell lines. Our results showed that miR-195 and -503 levels were significantly lower (*P* ≤ 0.05) in MKN45 cells than in AGS cells (Supplementary Fig. 4B). Taken together, these data suggest an involvement of miR-195 and -503 in the upregulation of *CHK1* mRNA in MKN45 cells.

### Prognostic Relevance of Chk1 Expression in gastric tumors

**Figure 5:**
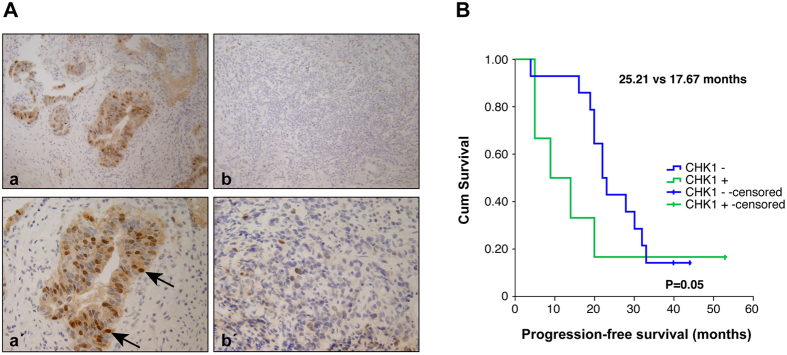
Nuclear Chk1 protein levels correlate with poor clinical outcome in human gastric cancer. (**A**) Immunohistochemical staining of Chk1 in representative carcinoma GC specimens: Nuclear positive and negative staining at 20 HPF (**a,b** respectively); nuclear positive and negative staining at 40HPF (**a**’,**b**’ respectively). The arrows point to strong Chk1 staining in the nucleus. (**B**) Kaplan-Meier curves of progression-free survival (PFS) in patients with high or low expression of nuclear Chk1 in their gastric tumors. Survival curves were statistically different when analyzed by the Breslow. IBM SPSS Statistics 22 software.

To assess the clinical significance of these findings, we studied the levels of Chk1 protein in a small cohort of patients. The samples were selected from patients that had received adjuvant therapy (Radiotherapy plus 5-FU) after surgery. The overall features of all 23 patients are summarized in Table 1. No significant association between Chk1 levels and age, sex, stage of the tumor, Lauren and Her2+ expression or PFS was observed in patients with GC (p > 0,05). However, our analysis detected 6 cases with positive Chk1 nuclear staining *versus* 14 cases that did not show any staining for Chk1 (Fig. 5A). Interestingly, we observed that patients with nuclear Chk1 accumulation tended to have shorter PFS than those with negative nuclear Chk1 staining (17,67 months vs 25,21 months; p = 0.059). The Kaplan-Meyer curve showed early differences in PFS between both populations (Fig. 5B). Altogether these results suggest that Chk1 can be considered as a putative biomarker for radiotherapy response in GC patients, since Chk1 protein level correlates with poor clinical outcome in human GC.

## Discussion

Combining chemotherapy with radiation improves outcome in GC, but this strategy comes with the price of an increased toxicity rate and furthermore, many of these tumors are resistant to radiation. To overcome this obstacle, it is crucial to identify the key determinants of radioresistance, since this will enable us to develop safer and more effective tumor radiosensitizers.

We have previously reported that CDDP-resistance in colorectal cancer cells correlates with high Chk1 levels^32^. Here, we have demonstrated a relationship between Chk1 expression and IR resistance in GC. Our results indicate that in GC cell lines, Chk1 is upregulated and specifically modulates sensitivity to radiation. This upregulation correlates with poor clinical prognosis in our patient cohort and can be explained by acquired resistance to IR. Taking into account that GC is generally diagnosed at advanced stages, it is difficult to recruit a large cohort of patients meeting our inclusion criteria (gastrectomy plus adjuvant therapy based on 5-FU plus IR). However, it is necessary now to reproduce our results in a larger cohort of patients, to confirm the implication of Chk1 on therapy response and also to clarify other clinical and pathological outcomes. Our data indicate that inhibition of Chk1 activity due to treatment with UCN-01, increases sensitivity to both BLM and IR. This evidence points to Chk1 as a good target in GC treatment. Unfortunately, clinical development of UCN-01 has been halted due to unfavorable pharmacology^46^. However, other inhibitors have been tested such as AZD7762 (a potent and selective ATP-competitive Chk1 kinase inhibitor), which has shown strong chemosensitizing activity when used alongside DNA-damaging agents. This has been evaluated both in *in vitro* and *in vivo* model systems^47^. Increasing evidence indicates that Chk1 inhibitors are able to synergize with antitumoral drugs in an specific molecular context, such as tumors with defects in the DNA damage repair pathway, or those overexpressing specific oncogenes^48^.

According with data from the literature, transcriptional regulation of Chk1 is controlled through p53 and E2F1^49^,^50^. In our experimental model radioresistance of MKN45 cells is not dependent on p53, since in both cell lines used in our study, p53 is wild type^37^. We corroborated that p53 is equally activated and follows the same kinetics in both cell lines after IR treatment. Our luciferase assays demonstrated that p53 regulates negatively the transcriptional activation of Chk1; moreover, we also confirmed that overexpression of p53 by transfection, leads to downregulation of Chk1 mRNA *in vitro*.

We cloned the Chk1 promoter region which contains different E2F binding sites^50^. The RB1-E2F1 pathway is crucial for the regulation of cell cycle progression and tumorigenesis. RB1 is a tumor suppressor gene frequently mutated or deleted in cancer, however in GC it is also amplified in an important percentage of samples^51^. Our results support a definitive role of RB1-E2F1 in the regulation of Chk1 transcription in GC. We suggest that in basal conditions E2F1 is sequestered by RB1, which is then unable to induce the expression of Chk1. This hypothesis is supported by our experiment with E1A oncoprotein, which binds and inhibits RB family members by disrupting E2F-RB1 interactions^52^, thus increasing Chk1 promoter activity, especially in MKN45 cells with higher levels of RB1. In addition, in response to E2F1 overexpression these cells are unable to increase Chk1’s mRNA.

Several studies have shown that both miR-15 and miR-497 families modulate multidrug resistance in GC cells by targeting BCl-2^53^,^54^,^55^,^56^. Furthermore, this family of miRNA controls Chk1 protein levels. For instance, overexpression of the miR-15a/b and miR-16 family affects the radiosensititivity of human breast cancer by regulating Chk1 and Wee1 proteins^43^. On the contrary, it has been shown that downregulation of the miR-15 family regulates CDDP sensitivity by increasing Chk1 levels^44^. Other studies have demonstrated that dowregulation of miR-424 contributes to cervical cancer progression via upregulation of its target gene Chk1^11^. Along these lines, our results show a significant downregulation of miR-195 and miR-503 expression in MKN45 radioresistant cells. Accordingly, NSCLC (non small cell lung carcinoma) shows lower miR-195 expression in the tumor than in adjacent tissues, and this lower expression has been associated with poorer overall survival^57^. However, in addition to our preliminary results, more experiments are needed to specifically confirm the impact of this family of microRNAs on Chk1’s expression and to define its influence on radioresistance. Nonetheless, the inverse correlation between microRNAs and Chk1 would be a promising parameter to consider in the clinical setting. In this regard, recent evidence confirms our findings in lung cancer^57^ which supports the universal character of our observations. However, other putative targets of those microRNA should be taken into account, and also their relationship with E2F1-RB^58^, in order to describe a possible feedback loop that could be regulating each member.

In summary, in this study we demonstrate that gastric tumors can be stratified into radiation resistant or sensitive, according to the status of Chk1. Chk1 protein levels could be modified by pRB-E2F1, p53 or miRNAs that seem to regulate its expression Thus, the miR-195/Chk1 axis may be used as a new biomarker tool to predict individual response to adjuvant radiotherapy. We previously described the possibility to sensitize advanced tumoral gastric cancer cells by adjuvant chemotherapy based on paclitaxel and cisplatin^59^, as an alternative to non-resecable tumors. The data presented here could mark a step forward allowing the design of improved therapeutic interventions for GC patients.

## Materials and Methods

### Cell lines

AGS and MKN45 human gastric adenocarcinoma cell lines were cultured in F12-Kaings and RPMI mediums respectively (Gibco), and supplemented with FBS (10% for AGS and 20% for MKN45). Cultures were maintained at 37 °C, 5% CO_2_ and 95% humidity. AGS and MKN45 are wild type for TP53^37^,^38^.

### Cloning

Genomic DNA was extracted from the colorectal cancer cell line HCT116 and the required sequence (−1823 –284) was cloned into a pGL3-enhanced luciferase plasmid vector. *See Supplementary M&M for details.*

### PCR

Total RNA was extracted using Tri-Reagent (Life technologies). Gene expression levels were assessed by Q-PCR, by using SYBR green-based chemistries for amplicon detection. For relative quantification (RQ) we used the delta-Ct method. Statistical analyses was performed for each gene by using a paired t-test to compare mean values, where p < 0.05 was considered significant.

### Reagents and Plasmid Vectors

Cisplatin, 5-Fluorouracile, Actinomycin D and UCN-01 were purchased from Sigma Aldrich. BLM was purchased from Calbiochem. pGL3 Basic and pGEMT easy (Promega), pSG5-HEY1, and pSG5 (Dr. B. Belandia), pCMV-E2F1 (Dra. A. Zubiaga), pcdna-p53DN (Dr. I Palmero), PCEFL-E1A, PEF1-p53 wt and pEF1 Dn (Dr. Sanchez-Prieto).

### Luciferase activity assay

Constructs carrying the luciferase gene were cotransfected with 1 ng of Renilla (transfecting control, 1:100) using lipofectamine 2000 (Invitrogen) in 24 well plates following the Manufacter’s instructions. 24 hours after transfection, transcriptional activity was quantified using the Dual-Luciferase® Reporter (DLR™) Assay System (Promega) using a Promega luminometer.

### Cell viability Clonogenic assay

Viability was determined using a MTS (Promega) staining method, as described^60^. To assess the effects of irradiation, cells were irradiated with different doses of Gy (0–8 Gy) using a ^137^Cs source (mark 1, model 30, JL. Shepherd & Associates San Fernando CA; Dose rate to 100 mm diameter samples is ~370 R/minute). 15 days after treatment, colonies containing more than 50 individual cells were counted using a microscope and survival fractions were quantified as described^61^.

### IC50 and Combination Index (CI)

IC50 were calculated by using the GraphPad Prism program. We used nonlinear regression to fit the data to the log (inhibitor) vs response (variable slope) curve.

Effects of BLM and UCN-01 combination on growth inhibition were analyzed by the Combination Index (CI) equation developed by Chou-Talalay^35^,^62^ using the CalcuSyn program (Biosoft, Cambridge, UK). The general equation for the classic isobologram is given by: CI = (D)1/(Dx)1 + (D)2/(Dx)2 where CI < 1 indicates synergism; CI = 1 indicates additive effect, and CI > 1 indicates antagonism; (Dx)1 and (Dx)2 in the denominators are the doses (or concentrations) of D1 (drug #1, for example, BLM) and D2 (drug #2, for example, UCN-01) alone that gives x% inhibition, whereas (D)1 and (D)2 in the numerators are the doses of D1 and D2 in combination that also inhibits x% The (Dx)1 and (Dx)2 can be readily calculated from the median-effect equation of Chou Dx = Dm[fa/(1-fa)]1/m where Dx is the median-effect dose, fa is the fraction affected, Dm is the median-effect dose signifying potency and m is the kinetic order signifying the shape of dose-effect curve.

### Western blotting

Twenty μg of protein per sample were loaded in SDS-PAGE in 10% (for E2F1,Chk1 and p53) or 8% (for Rb or PARP-1) polyacrylamide gels, and then transferred onto nitrocellulose membranes. Antibody dilutions were as follows: Chk1- 1:500 (sc-377231), E2F1- 1:1000 (sc-193), Rb- 1:200 (sc-102), PARP-1- 1:1000 (H-300: sc-25780), Cleaved Caspase-3 (Asp175) Antibody #9661 1:1000, p53 Antibody #9282 1:1000 (Cell signaling) in 5% fat free milk 0’05% TTBS. HA Antibody- 1:2000 (Boehringer mannheim), Flag antibody- 1:2000 (Sigma)

### Cell cycle analysis

Cell cycle analysis was performed as previously described^59^. *Supplementary M&M for details.*

### Viral transduction of target cells

Viral particles for infection were generated according to manufacturing instructions using GIPZ Lentiviral shRNA for CHK1 (Thermo Scientific Open Biosystems). *See Supplementary M&M for details.*

### miRNA analysis

RNA was purified from cultured cells using mirVana™ miRNA Isolation Kit (Ambion; Life Technologies). RT: TaqMan® MicroRNA Reverse Transcription Kit and TaqMan microRNA specific assays (Catalog #: 4427975 ID: 00104; ID: 000494: ID: 001973) were used to perform the RT and quantitative PCR (RT-qPCR) of triplicate samples. Real-time PCR was performed on an Applied Biosystems Step-one plus PCR (Life Technologies) following the manufacturer’s instructions. Total RNA input was normalized using RNU6B RNA as an endogenous control.

### Gene expression profile analysis

The miRNA expression data in a large set of GC patient samples^42^ was downloaded from the Gene Expression Omnibus (GEO, http://www.ncbi.nlm.nih.gov/geo/) and analyzed using custom R scripts for statistical programming http://www.r-project.org/“. Briefly, we compared probe values in each sample group (“normal”, “pretreatment”, “post-treatment”) using a Student’s t-test and the resulting p-values were adjusted for multiple testing by the Bonferroni method.

### Patients and tumor samples

Patients (n = 23) were recruited from the Oncology Department at the Infanta Sofía´s Hospital between 2008 and 2015. We selected those patients diagnosed with GC, who underwent radical surgery and received adjuvant therapy according to the MacDonald´s treatment guidelines (5-FU and radiation). The specimens were collected during tumor resection. Tissue samples were histologically confirmed as tumoral or non-tumoral tissues, and were stored at −80 °C until analysis. This study was approved by the Clinical Research Ethics Committee “IMDEA alimentacion” (code IMD: PI-010). All procedures were carried out in accordance with the approved guidelines and after informed consent was obtained from all subjects included in the study.

### Immunohistochemical analysis

Immunohistochemistry was performed in 3-μm sections of paraffin-embedded tissues. Samples were deparaffinized and rehydrated in water, after which antigen retrieval was carried out by incubation in EDTA solution. Endogenous peroxidase and non-specific antibody reactivity were blocked with peroxidase blocking reagent (Dako). The sections were then incubated with the Rabbit Monoclonal Antibody against Chk1 (TA300658. Origene Technologies). Detection was carried out by using Envision Plus Detection System (Dako). Negative controls were performed by replacing the primary antibody with goat serum. The slides were finally mounted with DPX mountant for microscopy (VWR Int). Immunohistochemical analysis was performed by the Pathology department’s staff at the same Hospital and the interpretation by a blinded expert pathologist. Chk1 nuclear staining was assessed as a percentage of surface showing positive signal, relative to the percentage of stained cells in the sample per surface area. A semi-quantitative score was assigned, as follows: 0: ≤10%; 1: 10–25%; 2: 25–50%; 3: ≥50%. Positive staining was therefore considered 1, 2 or 3.

## Additional Information

**How to cite this article**: Bargiela-Iparraguirre, J. *et al.* CHK1 expression in Gastric Cancer is modulated by p53 and RB1/E2F1: implications in chemo/radiotherapy response. *Sci. Rep.* **6**, 21519; doi: 10.1038/srep21519 (2016).

## Supplementary Material

Supplementary Information

## Figures and Tables

**Table 1:** The association of Chk1 nuclear expression with the clinicopathological characteristics of Gastric Cancer patients.

GC Characteristic		CHK1 Low	CHK1 High		
	n*	(%)	(%)	*X*^*2*^	*P*
SEX					
Male	19	68,5	31,5	1.709	0.539
Female	4	100	0		
AGE (mean)		63,3	74		0.389
STAGE					
I	3	100	0	2.841	0.417
II	7	57,1	42,9		
III	6	83,3	16,7		
IV	1	100	0		
LAUREN					
Intestinal	8	87,5	12,5	0.410	0.522
Difusse	8	75	25		
HER2/erb-b2					
Positive	2	50	50	0.647	0.421
Negative	17	70	30		
PFS (Months)					
≤18	5	40	60	4.752	0.063
>18	11	90	10		

Results from a total of 23 patient samples. Correlation between clinicopathological characteristics and Chk1 nuclear expression was assessed by *Chi*^*2*^ or *Fisher* exact test and *t-test* to compare the mean age between groups. Statistical significance was considered when p < 0.05, using IBM SPSS Statistics 22 software.

*Patients with missing clinicopathological information in their medical records.
