# Supplementary material for: CHK1 expression in Gastric Cancer is modulated by p53 and RB1/E2F1: implications in chemo/radiotherapy response
Source: Sci Rep. 2016 Feb 12;6:21519. doi: 10.1038/srep21519 (PMC4751465; doi:10.1038/srep21519)
Supplement: Supplementary Information [file srep21519-s1.pdf]

CHK1 expression in Gastric Cancer is modulated by p53 and RB1/E2F1: implication in chemo/radiotherapy response.

Bargiela-Iparraguirre J[1†], Prado-Marchal L[1†], Fernandez-Fuente M[2], Gutierrez-González A [1], Moreno-Rubio J [3,4], Muñoz-Fernandez M [5], Sereno M [3], Sanchez-Prieto, R [6,7], Perona R [1,8,9] and Sanchez-Perez I[1,6,7,8,9 *]

[1] Dpto.Bioquímica. Fac. Medicina. Instituto de Investigaciones Biomédicas Madrid CSIC-UAM; Madrid, Spain
[2] The Royal Veterinary College. University of London; London, UK
[3] Medical Oncology Department, Infanta Sofía University Hospital, San Sebastian de los Reyes, Madrid, 28702; Spain
[4]IMDEA-Food Institute, CEI UAM+CSIC, Madrid, Spain
[5]Pathology Department, Infanta Sofía University Hospital, San Sebastián de los Reyes, 28702, Madrid
[6] Unidad de Medicina Molecular, laboratorio de Oncología ,CRIB/FPCYT C-LM. Universidad de Castilla-La Mancha, Av. Almansa 14, 02006, Albacete, Spain
[7]Unidad asociada de Biomedicina UCLM-CSIC[5 6]
[8] CIBER for Rare Diseases (CIBERER); Valencia, Spain
[9]Biomarkers and Experimental Therapeutics Group; IdiPAZ; University Hospital La Paz; Madrid, Spain

Supplementary M&M

Cloning and PCR.

The promoter sequences of interest were amplified by PCR and cloned into the intermediate vector pGEMT-easy (Promega). Fidelity was verified by sequencing. Promoter sequences were then subcloned upstream of the luciferase gene, into the final vector pGL3-Luciferase Basic (Promega), using specific restriction sites for BglII and MluI. The following primer pairs were used:

F0: Fw: 5' GGACGCGTAAGCCATTCTCCTGCCTCGC-3'

F1: Fw 5' GGACGCGTGGTGCAGCCTTTCAGGCCCA-3'

F2: Fw 5'- GGACGCGTGCGCTGTCTTGCTTTACGGC-3'

F3: Fw: 5'- GGACGCGTAGAAGGAGTTCGGGGTCTAG-3'

Rv: 5'- GGAGATCTCCGGCGAACGACTGGGGAAG-3'

Primers used for the short isoform of CHEK1:

1F:5'-GACTGGGACTTGGTGCAAAC-3'

2F: 5'CTGAAGAAGCAGTCGCAGTG-3'

1R: 5'-GCAGGAAGCCAAATCTTCTG-3'

2R: 5'-TGGGAGACTCTGACACACCA-3'

Viral transduction of target cells.

Briefly, 293T cells (4.5 x 10^6 cells/plate) were transfected using lipofectamine 2000 (Invitrogen) with 15 µg of shCHEK1, 7 µg of envelope plasmid (VSV-G), and 7 µg of Helper plasmid (pCD/NL-BH). The supernatants were recovered 48h and 72h after transfection and frozen in small aliquots at -80ºC until used. Transduction was carried out using 5 x 10^5 cells per well in a 6-well plate. 48 hours post-transduction cells were examined microscopically for the presence of GFP-reporter expression as an indicator of transduction efficiency. 72h after, cells were assayed for changes in gene expression by quantitative/real-time PCR (QPCR), compared to non-silencing shRNA.

Cell cycle analysis.

Adherent and non-adherent cells were harvested and fixed overnight in 70% ethanol in phosphate-buffered saline (PBS). For DNA content analysis, cells were centrifuged and resuspended in PBS containing 1 µg/ml RNase (Qiagen Ltd., Crawley, UK) and 25 µg/ml propidium iodide (Sigma-Alcrich), then incubated at room temperature for 30 min, and finally analyzed using a Becton Dickinson Flow Cytometer (Cowley, UK). Data were plotted using Cell Quest software, with 10,000 events analyzed per sample.

Supplementary figure legends

Supplementary Fig. 1

(A) Representative images of a Clonogenic assay of AGS and MKN45 cells at the end of the experiment (13 days) after treatment with IR (0-8 Gy) or BLM (0-7,5 µg/ml). (**B)** *CHK1* mRNA expression in non tumoral cells (Hs 738.St/Int) and GC cell lines (AGS, MKN45, KatoIII and Hs746t) was extracted from deposited CHK1 Copy Number data in Rothenberg Cell Line data sets, analyzed in Oncomine and presented as bars. (C) Schematic representation of the primers used to amplify the two isoforms of *CHK1* (adapted from [1]) by RT-PCR. The picture represents the amplicons detected with each primer set. Only one amplicon was detected using primers set 1 (lane1), whereas two amplicons were obtained when using primer sets 2 or 3. (Lanes 2 and 3). Lane 4 represents the amplification of our endogenous control GAPDH.

Supplementary Fig. 2

(A) UCN-01 (100 nmol/L) was added 30 minutes before bleomycin (10 ug / ml) treatment. Cells were harvested 3 hours later, and phosphorylation of Chk1-Ser296 was studied by WB. (B) mRNA was extracted 72 h after transduction and Q-PCR showed that Chk1 is depleted during the experimental process.

Supplementary Fig. 3

(A) Schematic representation of the constructs used in our transfection experiments. The sequence -1843-287 contains promoter elements and the graph shows the predicted (Transfact tool) transcription factors (TF) that bind to the cloned sequence. (B) Cell lines were co-transfected with 250 and 500 ng of F0-pGL3 plasmid a luciferase reporter construct carrying the Chk1 promoter fragment (-1834-234). The graph shows the expression levels relative to the empty PGL3 vector and normalized by Renilla. Bars represent the average of at least three experiments performed in triplicate (*p<0,05).(C) AGS and MKN45 cells were treated with IR (8 G). Cells were harvested at the indicated times, and p53 stabilization was analyzed by using a specific antibody against p53. α-Tubulin was used as a loading control. (D) AGS and MKN45 cells were transfected as indicated in Figure 3, and mRNAs were extracted 24h after transfection. Chk1 levels were quantified by RT-QPCR. The graph represents the relative levels of Chk1, compared with normal control cells. Transfection of p53 and Hey1 was followed by western blot using a specific antibody against the flag epitope (Hey1) or HA epitope ($p53^{WT}$ or $p53^{DN}$). Tubulin was used as a loading control.

Supplementary Fig. 4

CHK1 is controlled at posttranscriptional level by miRNA-195 and miRNA-503. (A) Cells were treated with Act D (1 µg/ml) for the indicated periods (0-9 h) and *CHK1* mRNA levels were determined by semi-quantitative RT-PCR. Data indicate the intensity of *CHK1* bands normalized with GAPDH, and are presented as mean ± SD of three independent experiments (*$p < 0.05$). (B) Table: differentially expressed miRNAs in GC and normal stomach epithelium with *CHK1* as a potential target. Alignment of the selected miRNAs with the 3'UTR sequence of *CHK1*. Fold changes of miR-195 and miR-503 expression levels in AGS and MKN45 cells. Gene expression is presented as the expression value relative to that of the U6 endogenous control. The experiment was performed in triplicate. Data are presented as mean ± standard deviation (SD).

1 Pabla, N., Bhatt, K. & Dong, Z. Checkpoint kinase 1 (Chk1)-short is a splice variant and endogenous inhibitor of Chk1 that regulates cell cycle and DNA damage checkpoints. *Proc Natl Acad Sci U S A* **109**, 197-202, doi:10.1073/pnas.1104767109 (2012).

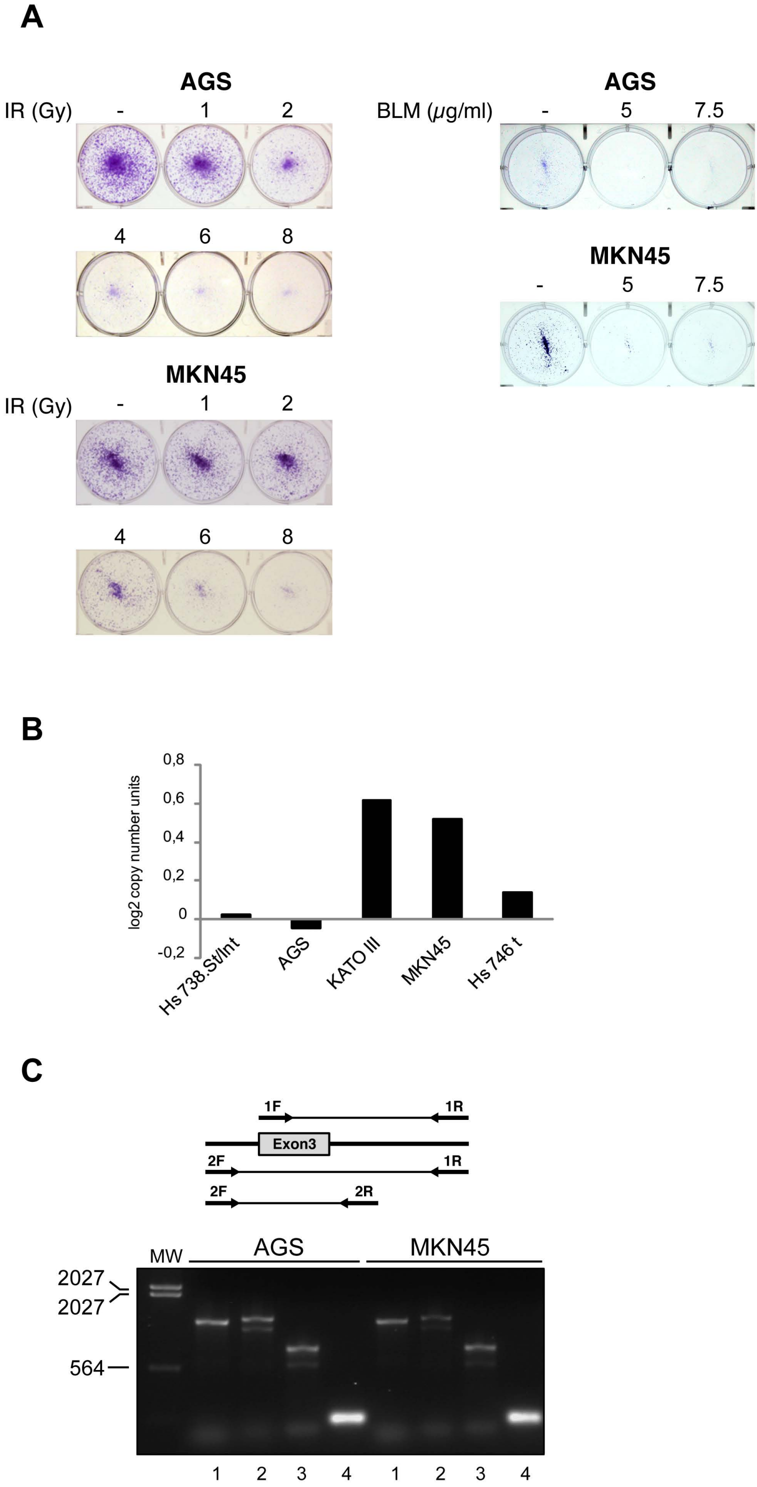
A
AGS
IR (Gy)
-
1
2
4
6
8
AGS
BLM (μg/ml)
-
5
7.5
MKN45
-
5
7.5
MKN45
IR (Gy)
-
1
2
4
6
8
B
log2 copy number units
0,8
0,6
0,4
0,2
0
-0,2
Hs 738.St/Int
AGS
KATO III
MKN45
Hs 746 t
C
1F
1R
Exon3
2F
1R
2F
2R
MW
AGS
MKN45
2027
2027
564
1
2
3
4
1
2
3
4

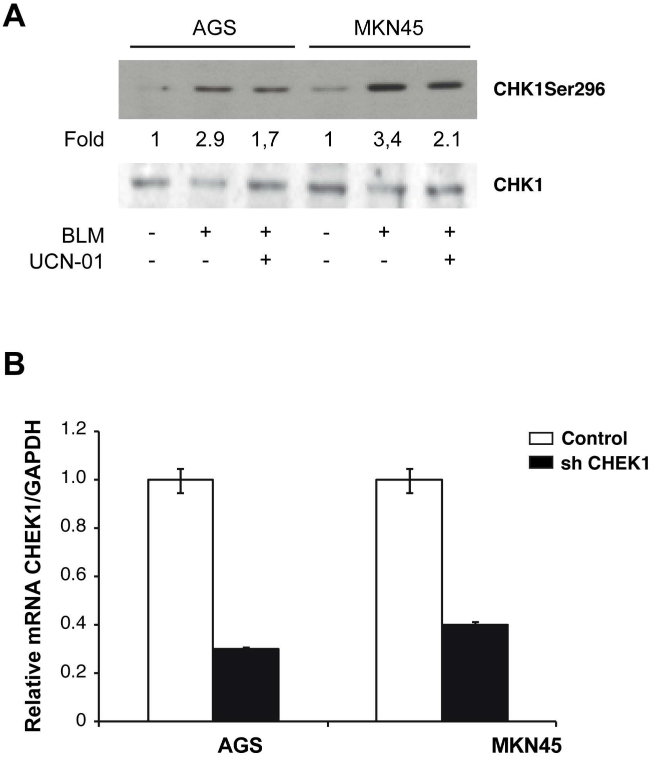
A
AGS
MKN45
CHK1Ser296
Fold 1 2.9 1,7 1 3,4 2.1
CHK1
BLM - + + - + +
UCN-01 - - + - - +
B
Relative mRNA CHEK1/GAPDH
1.2
1.0
0.8
0.6
0.4
0.2
0
Control
sh CHEK1
AGS
MKN45

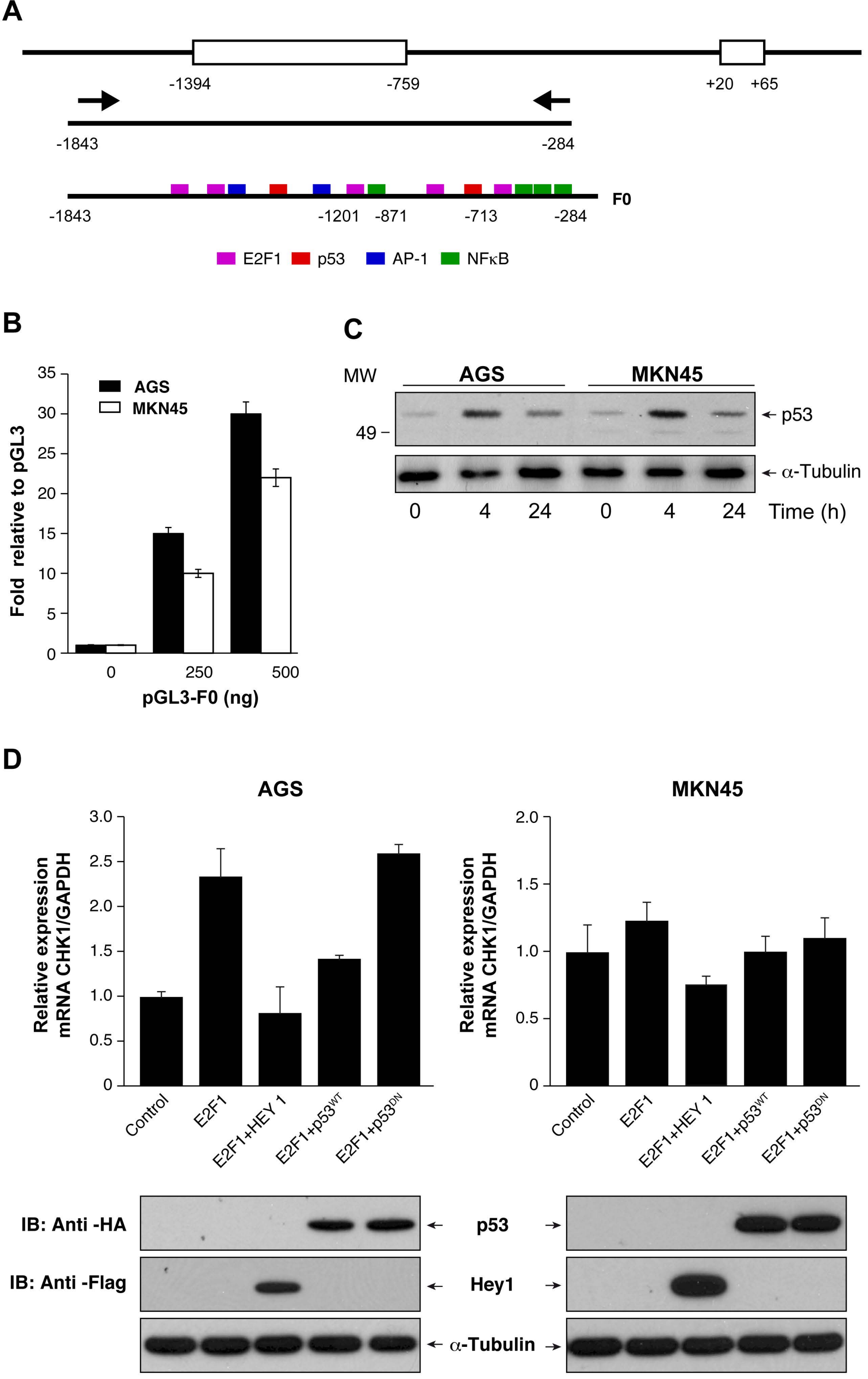
A
-1394
-759
+20
+65
-1843
-284
F0
-1201
-871
-713
E2F1
p53
AP-1
NFκB
B
AGS
MKN45
Fold relative to pGL3
pGL3-F0 (ng)
C
MW
49
p53
α-Tubulin
Time (h)
D
Relative expression mRNA CHK1/GAPDH
Control
E2F1
E2F1+HEY 1
E2F1+p53WT
E2F1+p53DN
IB: Anti -HA
IB: Anti -Flag
Hey1

A

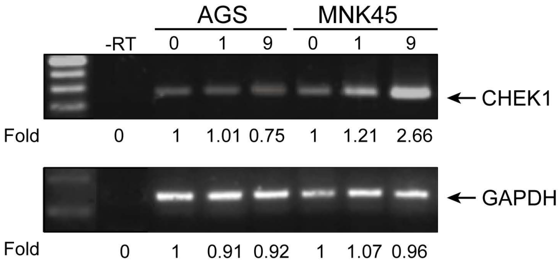

B

siRNA target	Predicted targets	p-Value	FDR
CHEK1	hsa-miR-497	7.11E-05	1.71E-01
CHEK1	hsa-miR-195	2.73E-06	1.29E-02
CHEK1	hsa-miR-16	0.580	1.00E-00
CHEK1	hsa-miR-503	8.89E-06	2.04E-02
CHEK1	hsa-miR-15	0.094	1.00E-00

hsa-miR-195/CHEK1 Alignment

```
   3´  cggUUAUAAAGACACGACGAu      5´  hsa-miR-195
          |||||    ||||||||
28:5´  gugAAUAU---AGUGCUGCUa      3´  CHEK1
```

hsa-miR-503/CHEK1 Alignment

```
   3´  gacgucuugacaaggGCGACGAu  5´  hsa-miR-503
                      |||||||
23:5´  uccuggugaauauagUGCUGCUa  3´  CHEK1
```

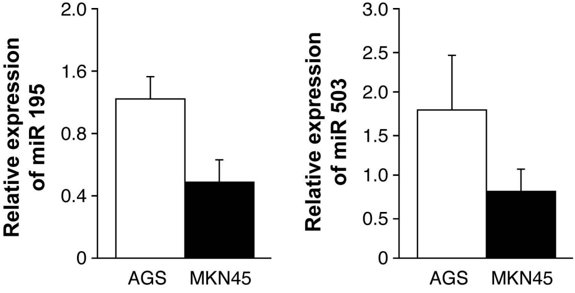

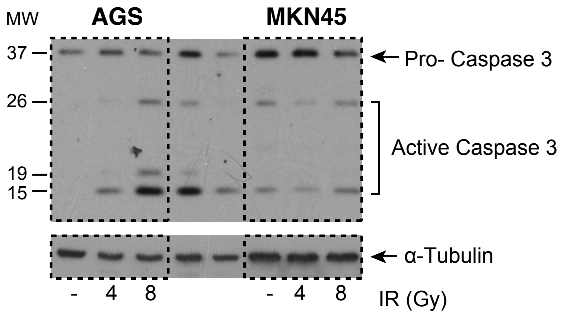
MW
AGS
MKN45
37
26
19
15
Pro- Caspase 3
Active Caspase 3
α-Tubulin
- 4 8
- 4 8
IR (Gy)
